# Biomorphometric and hematobiochemical alterations in the juvenile african catfish *Clarias gariepinus* exposed to propranolol

**DOI:** 10.1186/s40850-024-00196-x

**Published:** 2024-06-20

**Authors:** Temitope D. Melefa, Funmilayo F. Hinmikaiye, Felix A. Andong, Daniel E. Echude, Daoud Ali, Saud Alarifi, Priscilla Nkeonye Abara, Christopher. D. Nwani

**Affiliations:** 1 Department of Zoology and Environmental Biology, University of Nigeria, Nsukka, Nigeria; 2 Department of Zoology, College of Science, King Saud University, 11451 Riyadh, PO Box 2455, Saudi Arabia; 3 Department of Biology, Federal University of Technology, Owerri, Nigeria

**Keywords:** African freshwater catfish, Toxicity, Physiology, Propranolol

## Abstract

Propranolol (PRO) is a beta-blocker drug used for the treatment of anxiety, chest pain, migraine and tremors. The present study investigated whether sublethal concentrations of PRO have effects on the body condition, biochemistry, and hematology of *Clarias gariepinus* juveniles. The 96-h median lethal concentration (LC_50_) of the drug, which was established through an acute toxicity study, was 9.48 mg/L. Based on these values, the fish were exposed for 21 days to the control and sublethal concentrations of 1.90, 0.95, and 0.63 mg/L, which are equivalent to the 1/5th, 1/10th, and 1/20th of the LC_50_ of PRO, respectively. After 21 days of exposure, the fish were removed from the toxicant and kept in toxicant-free water for 7 days to recover. The standard length and body weight of each fish were measured after each exposure period. The condition factor (CF) and hepatosomatic index (HSI) were not significantly affected by the drug. The red blood cell (RBC) count, hemoglobin (Hb) count and packed cell volume (PCV) decreased from day 7 to 21 at the tested concentrations, while the white blood cell (WBC) count significantly increased. There were alterations in the mean corpuscular volume (MCV), mean corpuscular hemoglobin concentration (MCHC), and mean corpuscular hemoglobin (MCH) in the exposed groups compared to those in the control group. When neutrophil counts increased, the lymphocyte counts decreased, but the monocyte, basophil cell and eosinophil cell counts were not affected. Among the liver enzymes, only aspartate aminotransferase was significantly stimulated in the groups that were exposed to the drug. The protein and glucose levels of fish exposed to the drug decreased. Most of the studied parameters returned to their original values after the 7-day recovery period. The information provided in the current study will be helpful in the monitoring of PRO contamination in aquatic environments.

**Supplementary Information:**

The online version contains supplementary material available at 10.1186/s40850-024-00196-x.

## Introduction

Pharmaceuticals are environmental pollutants that are used extensively in human and veterinary medicine. These contaminants are categorized as emerging organic contaminants [1, 2]. The presence of these compounds in various aquatic environments at concentrations that can adversely affect aquatic organisms has increased recently because they are widely and frequently used [3, 4]. Beta and adrenergic receptor blockers are drugs that act on the sympathetic nervous system by blocking beta1 and 2 adrenergic receptors in the central nervous system to reduce the effects of adrenaline and noradrenaline [5]. Propranolol is a nonselective beta-adrenergic receptor blocker (beta-blocker) used to treat heart-related diseases and anxiety [6]. The drug PRO is a pharmaceutical used for the treatment of anxiety, chest pain, migraine and various types of tremors [5]. They are commonly found in aquatic environments and has a half-life of approximately 6 h [7]. It is found in wastewater, rivers and estuaries around the world at various concentrations (in ng/L) [3, 8–10]. In Nigeria, PRO values of 1–12 ng/L have been reported in some rivers in the Lagos area [11, 12]. Although the concentrations of some pharmaceuticals in freshwater are low, many of them and their metabolites are biologically active and can harm aquatic organisms that are not their intended targets [13]. Pharmaceuticals are known to have negative effects on organisms other than their intended targets, and exposure (both acute and chronic) can cause irreversible changes in important physiological processes in specific organisms [14–16]. According to Capolupo et al. [17], PRO significantly decreases fertilization in sea urchins. Additionally, after 96 h of exposure to PRO, fewer seabream larvae survived. Diatoms exposed to PRO resulted in oxidative damage, a significant decrease in the generation of oxygen and an increase in heterotrophic mitochondrial function [7]. Numerous species have been studied to determine how drugs affect nontarget organisms, particularly fish [18–23]. Hematological and biochemical parameters have been employed in understanding the effects of xenobiotics in aquatic organisms [21, 24, 25].

The African freshwater catfish *Clarias gariepinus* was chosen for this project due to its widespread distribution in Nigeria and most African countries. The fish is readily available year-round and adapts well to laboratory conditions [26]. Although PRO is found in various aquatic compartments, there are still few ecotoxicological studies on the impact of this drug on tropical species of nontarget organisms, as different species may respond differently to contaminants. Chronic exposure to PRO may lead to alterations in normal physiological processes in nontarget aquatic organisms, especially fish, as they are continually exposed to these contaminants. To the best of our knowledge, no research has been conducted on the ecotoxicity of PRO in *C. gariepinus*. In light of these findings, this study investigated the morphological and hemobiochemical changes in *C. gariepinus* exposed to PRO.

## Materials and methods

Three hundred and sixty (360) juveniles of the freshwater African catfish *C. gariepinus* (mean ± standard deviation of length 27.36 ± 0.23 cm and weight 197.39 ± 2.34 g) were purchased from a Freedom Fish Farm Nsukka, Nigeria and acclimated in a 1000 L capacity cement pond for 14 days. Water supplied to the ponds in the wet laboratory was obtained from the central university waterwork system. The mean (± standard deviation) temperature of the pond water and dissolved oxygen concentration were 26.04 ± 0.08 $\text{?}$and 8.84 mg/L, respectively. The study was conducted under a natural photoperiod of 14:10 light and dark cycles prevalent in Nuskka, Nigeria, when the research was conducted. The fish were fed a commercial diet daily (Coppens commercial feed of 4 mm, Coppens International Helmond Netherlands). Propranolol (PRO) tablet (AstraZeneca, USA) was used with 40 mg of propranolol as the active ingredient. A stock solution of the drug was prepared by dissolving a commercial tablet containing 40 mg of PRO (as the active ingredient) in 1 L of the stock solution. The different concentrations used for the study were prepared by serial dilution of the stock.

### Design of experiments for acute exposure

A range finding test was carried out, and the appropriate range of PRO concentrations (7 and 15 mg/L) that can cause percentage mortalities of 0% and 100%, respectively, was selected. From the obtained range values, ten [10] fish each from the acclimatized batch were then exposed to five (7, 9, 11, 13, and 15 mg/L) nominal concentrations of PRO and a control in glass aquaria (60 × 30 × 30 cm) containing 10 L of water in a static system. The experimental setup and control were replicated three times for robust statistical analysis. The fish were not fed during the acute toxicity studies to avoid possible contamination of the test water. At intervals of 24, 48, 72, and 96 h, the percentages of fish that died or survived in the control and treatment groups were noted. Dead fish were removed with plastic forceps immediately before they were removed to prevent deterioration of the test media. The fish were regarded as dead when their operculum stopped beating. The physicochemical characteristics of the test water were examined weekly [27], and the temperature, dissolved oxygen concentration, pH, conductivity and alkalinity were 26.2$\pm 0.50 \text{?}$, 6.9 ± 0.04 mg/L, 7.4 ± 0.02, 247.5 ± 4.89 µSC/m and 92.0 ± 3.20 mg/_L CO3,_ respectively. Probit analysis was calculated using SPSS 23.0.

### Sublethal exposure experiment

According to the probit analysis, the 96-h LC_50_ value of PRO in *C. gariepinus* was 9.48 mg/L. Based on these findings, three sublethal nominal concentrations of 1.90, 0.95, and 0.63 mg/L corresponding to the 1/5th, 1/10th, and 1/15th of the 96 h LC_50,_ respectively, were estimated and used for the in vivo experiment in fresh aquaria. The in vivo tests involved 120 acclimated fish from the batch. There were four treatment groups for fish. The first group received an exposure of 1.90 mg/L, the second group received 0.95 mg/L, the third group received 0.63 mg/L, and the fourth group received only nonchlorinated tap water as a control in a static experimental set-up. Each of the experiments was replicated three times, and 10 fish were set in aquaria containing 10 L of nonchlorinated water. The test solution was renewed every other day to maintain the concentration of the drug. No deaths were recorded in either the experimental or control groups. The fish were exposed to propranolol for 21 days and subsequently removed and transferred to drug-free water in a similar set up for 7 days to recover from the effects of the drug. To prevent starvation and subsequent effects, the fish were fed approximately 3% of their total body weight an hour before the test solution was renewed.

### Blood sampling and preparation

Blood was collected from two fish from each replicate tank in the treatment and control groups on days 1, 7, 14, 21, and 28 (after 7 days of recovery). Prior to blood collection, each fish was anesthetized with tricaine methanesulfonate (100 mg/L) to reduce stress [28]. To estimate the hematological parameters, approximately 0.5 ml of blood was drawn from the caudal vein using a heparinized syringe and preserved in small EDTA bottles at 4 °C. The blood samples obtained were divided into two parts. One part was used for estimation of the hematological parameters, while the second part was centrifuged at 10,000 × g at 4 $\text{?}$ for 20 min and used to estimate plasma glucose, protein, alanine aminotransferase (ALT), aspartate aminotransferase (AST) and alkaline phosphatase (ALP) levels.

### Hematological parameters

Using an advanced microscope Neubauer counter and Toison’s solution as the blood diluent, the red blood cell (RBC) count was calculated [29]. Turk’s solution was used as the blood diluent, while a Neubauer microscopic counter was used to calculate the white blood cell (WBC) count. The numbers of neutrophils, monocytes, lymphocytes, basophils and eosinophils in the blood smears were counted [30]. The hemoglobin (Hb) concentration in the blood was measured using the cyanmethemoglobin method [31]. By centrifuging the blood for 5 min at 1 400 × g in heparinized glass capillaries using a micro hematocrit centrifuge, the packed cell volume (PCV) was calculated. The PCV, Hb, and RBC were used to calculate the following erythrocyte indices: mean cell volume (MCV), mean cell hemoglobin volume (MCH), and mean cell hemoglobin concentration (MCHC) [32].

$$ \text{M}\text{C}\text{V} (\text{f}\text{l}/\text{c}\text{e}\text{l}\text{l}) =\frac{\text{P}\text{C}\text{V} \left(\text{\%}\right) \text{X} 10}{\text{R}\text{B}\text{C} \text{c}\text{o}\text{u}\text{n}\text{t} \text{i}\text{n} \text{m}\text{i}\text{l}\text{l}\text{i}\text{o}\text{n}/\text{m}\text{m}3}$$

$$ \text{M}\text{C}\text{H}(\text{p}\text{g}/\text{c}\text{e}\text{l}\text{l}) =\frac{\text{H}\text{b} \left(\text{g}/\text{d}\text{l}\right) \text{X} 10}{\text{R}\text{B}\text{C} \text{c}\text{o}\text{u}\text{n}\text{t} \text{i}\text{n} \text{m}\text{i}\text{l}\text{l}\text{i}\text{o}\text{n}/\text{m}\text{m}3}$$

$$ \text{M}\text{C}\text{H}\text{C} (\text{g}/\text{d}\text{l}) =\frac{\text{H}\text{b} \left(\text{g}/\text{d}\text{l}\right) \text{X} 10}{\text{P}\text{C}\text{V} \left(\text{\%}\right)\text{X} 100}$$

### Biochemical parameters

*The methods of* Reitman and Frankel [33] were used to measure the levels of plasma ALT and AST, while the levels of protein and glucose were determined following the methods of Cooper and Mcdaniel [34] and Lowry et al. [35], respectively.

### Morphometric indices

At each exposure sample point, each fish’s standard length and body weight were measured. Blood samples of approximately 0.5 ml were taken, after which the fish were dissected, and the liver was examined and weighed. Using the methods of White and Fletcher [36], the CF and HSI were computed as follows:

CF = Body weight (g) ⁄ Standard length (cm) ^3^ × 100.

HSI = Liver weight (g) × 100.

### Statistical analysis

SPSS version 23.0 (IBM Corporation, Armonk, USA) was used for statistical analysis. A generalized linear model (glm) coupled with two-way analysis of variance (ANOVA) was used to assess the effects of the exposure on the variables of interest. The drug concentration and duration of exposure were fixed factors in the models, while the dependent variables were entered as individual hematological, biochemical and morphometric parameters (i.e., univariate models). The significant drug concentration, exposure duration or interaction effects from the omnibus tests were assessed by the post hoc test, Duncan’s multiple range test. The effect size was computed as the partial eta (η_p_^2^) in the glm models. P values ≤ 0.05 were regarded as significant.

## Results

### Morphological indices

The body weights, condition factor (CF) and hepatosomatic index (HSI) of *C. gariepinus* following 21 days of exposure to PRO and the 7-day recovery period are presented in Table 1. The CF and HSI were not significantly affected by the drug. CF appeared to decrease progressively with prolongation of the setup. The drop was not due to the drug.

**Table 1:** Body weight, condition factor (CF) and hepatosomatic index (HSI) of *Clarias gariepinus* (*n* = 6) on 21-day exposure to propranolol and 7-day post withdrawal

Parameter	Conc. (mg/L)	Duration (day)				
		1	7	14	21	7-d withdrawal
Weight (g)	Control	54.67 ± 27.15^a1^	53.67 ± 7.10^a1^	50.33 ± 3.22^a1^	48.00 ± 8.54^a1^	49.00 ± 1.00^b1^
	1.90	49.33 ± 6.11^a1^	53.00 ± 1.00^a1^	44.33 ± 2.52^a1^	44.33 ± 14.05^a1^	50.33 ± 4.51^a1^
	0.95	53.67 ± 10.12^a1^	52.67 ± 8.08^a1^	48.67 ± 1.16^a1^	42.33 ± 1.53^a1^	51.00 ± 5.57^a1^
	0.63	51.33 ± 30.93^a1^	41.67 ± 6.66^a1^	47.33 ± 15.14^a1^	43.00 ± 1.00^a1^	54.33 ± 0.58^a1^
CF	Control	0.70 ± 0.07^a1^	0.71 ± 0.05^a1^	0.63 ± 0.05^a1^	0.67 ± 0.17^a1^	0.54 ± 0.08^a1^
	1.90	0.70 ± 0.17^a1^	0.69 ± 0.02^a1^	0.72 ± 0.09^a1^	0.57 ± 0.02^a1^	0.50 ± 0.08^a1^
	0.95	0.67 ± 0.07^a1^	0.66 ± 0.05^a1^	0.64 ± 0.08^a1^	0.70 ± 0.11^a1^	0.58 ± 0.06^a1^
	0.63	0.54 ± 0.17^a1^	0.71 ± 0.12^a1^	0.68 ± 0.10^a1^	0.60 ± 0.02^a1^	0.62 ± 0.05^a1^
HSI	Control	1.28 ± 0.20^a1^	1.15 ± 0.06^a1^	1.09 ± 0.29^a1^	1.18 ± 0.29^a1^	1.18 ± 0.16^a1^
	1.90	1.32 ± 0.28^a1^	1.21 ± 0.06^a1^	1.26 ± 0.14^a1^	1.10 ± 0.08^a1^	1.36 ± 0.14^a1^
	0.95	1.18 ± 0.12^a1^	1.40 ± 0.18^a1^	1.28 ± 0.04^a1^	1.27 ± 0.08^a1^	1.20 ± 0.29^a1^
	0.63	1.51 ± 0.06^a1^	1.14 ± 0.10^a1^	1.22 ± 0.05^a1^	1.13 ± 0.07^a1^	1.05 ± 0.18^a1^

Values as mean ± standard deviation, values with different alphabet superscript along a column for each parameter were significantly different; while those with different numeric superscript across a row were significantly different (*p* < 0.05)

### Hematological parameters

The effects of PRO on the hematological parameters PCV, Hb, RBC and WBC are summarized in Table 2. There was a concentration-dependent significant decrease in the PCV in *C. gariepinus* exposed to PRO from day 7 to 21. However, the PCV was significantly elevated after the 7-day recovery period. The Hb concentrations significantly decreased in the fish exposed to all concentrations of PRO throughout the duration of the exposure, except on day 1. After 7 days of recovery, Hb levels were elevated in the fish at all concentrations of PRO. There was generally a decrease in RBC levels in the fish exposed to various concentrations of the drug on days 7 to 21 compared with those in the control group. However, significant differences were observed only on day 21 postexposure to various PRO concentrations. However, the RBC values increased in the fish at all concentrations during the 7–day recovery period. There was a concentration- and duration-dependent increase in WBC values in *C. gariepinus* exposed to PRO. After 7 days of recovery, the WBC values were still elevated.

**Table 2:** Packed cell volume, haemoglobin concentration, red blood cell count and white blood cell count of *Clarias gariepinus* (*n* = 6) exposed to propranolol

Parameter	Conc. (mg/L)	Duration (day)				
		1	7	14	21	7-withdrawal
PCV (%)	Control	30.50 ± 0.50^b1^	30.50 ± 1.50^a1^	29.00 ± 1.00^a1^	33.00 ± 1.00^a1^	30.50 ± 0.50^a1^
	0.63	33.00 ± 1.00^a2^	27.00 ± 0.50^b1^	26.50 ± 0.50^b1^	26.00 ± 2.00^c1^	31.00 ± 3.00^a2^
	0.95	30.50 ± 0.50^b2^	27.00 ± 1.00^b1^	28.50 ± 0.50^a1^	25.50 ± 0.50^c1^	30.50 ± 0.50^a2^
	1.90	29.00 ± 1.00^b1^	26.50 ± 2.50^c1^	28.50 ± 1.50^a1^	29.00 ± 0.50^b1^	33.00 ± 1.00^a1^
Hb (g/dl)	Control	8.30 ± 0.10^a1^	9.75 ± 0.00^a2^	9.70 ± 0.00^a2^	10.02 ± 0.41^a2^	8.46 ± 0.18^a12^
	0.63	8.30 ± 0.10^a1^	8.30 ± 0.10^b1^	8.30 ± 0.10^b1^	8.35 ± 0.87^b1^	9.43 ± 0.22^a1^
	0.95	8.50 ± 0.30^a1^	8.45 ± 0.20^b1^	8.60 ± 0.40^b1^	8.79 ± 0.13^b1^	8.82 ± 0.40^a1^
	1.90	9.15 ± 0.15^a1^	8.45 ± 0.20^b1^	8.30 ± 0.10^b1^	8.17 ± 0.45^b1^	9.32 ± 0.10^a1^
RBC (10^12^/L)	Control	9.54 ± 0.12^a1^	10.38 ± 0.24^a1^	10.46 ± 0.21^a1^	8.85 ± 0.45^a1^	9.35 ± 0.35^a1^
	0.63	9.61 ± 0.03^a2^	9.52 ± 0.10^a2^	8.66 ± 0.45^b1^	9.05 ± 0.65^a2^	9.50 ± 0.10^a1^
	0.95	9.52 ± 0.11^a1^	8.98 ± 0.34^a1^	8.42 ± 0.22^b1^	8.20 ± 0.50^a1^	8.50 ± 0.30^a1^
	1.90	9.82 ± 0.61^a2^	9.88 ± 0.64^a2^	8.34 ± 0.08^b1^	8.30 ± 0.10^a1^	8.75 ± 0.55^a1^
WBC (10^6^/L)	Control	8700 ± 100^a1^	8850 ± 350^c1^	8800 ± 400^b1^	7950 ± 150^b1^	8350 ± 150^a1^
	0.63	8300 ± 100^a1^	11,100 ± 700^b2^	12,800 ± 800^a2^	10,600 ± 200^a2^	9950 ± 550^a2^
	0.95	8850 ± 350^a1^	10,400 ± 200^b2^	12,500 ± 100^a2^	11,750 ± 150^a2^	8400 ± 200^a1^
	1.90	8800 ± 300^a1^	10,700 ± 450^a2^	12,100 ± 900^a2^	10,800 ± 400^a2^	8850 ± 250^a1^

Values with different alphabet superscript along a column for organ type were significantly different for drug concentrations, while values with different numeric superscript across a row were significantly different for durations, (*p* < 0.05). 7-w = 7 day withdrawal

**Fig. 1:**
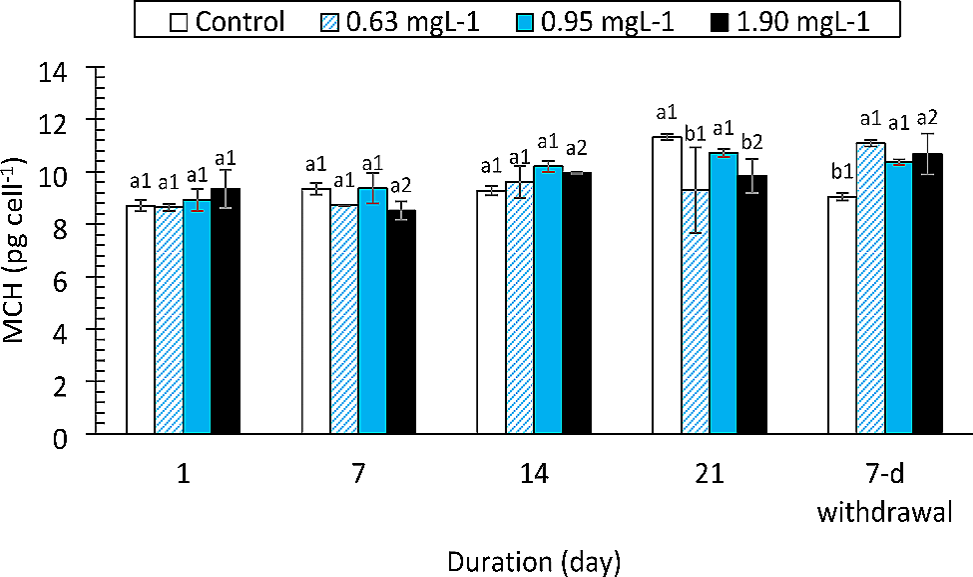
Mean corpuscular haemoglobin (MCH) of *Clarias gariepinus* (*n* = 6) exposed to propranolol. Bars with different alphabet label for a given day were significantly different for drug concentrations, while bars with different numeric label across were significantly different for durations (*p* < 0.05)

**Fig. 2:**
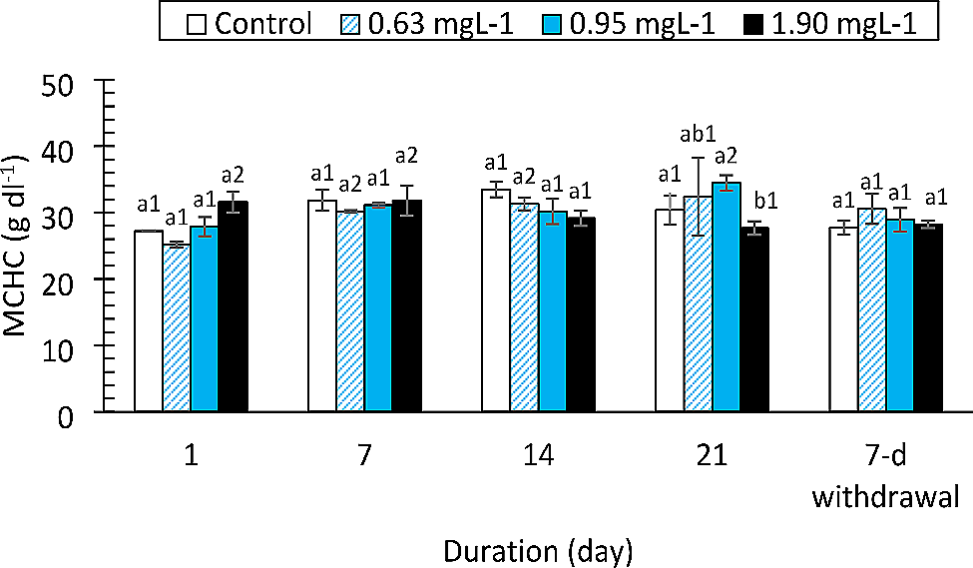
Mean corpuscular haemoglobin concentration (MCHC) of *Clarias gariepinus* (*n* = 6) exposed to propranolol. Bars with different alphabet label for a given day were significantly different for drug concentrations, while bars with different numeric label across were significantly different for durations (*p* < 0.05)

The effects of the drugs on MCH, MCHC and MCV are shown in Figs. 1 and 2 and S1, respectively. There were mixed trends in the MCH and MCHC values during the exposure periods. No significant differences were observed in the changes in the MCV at any of the concentrations during the exposure period. The effect sizes for changes in all three hematological indices due to drug concentrations were > 14% (η_p_^2^ = 0.100, 0.037, 0.137).

Sublethal concentrations of PRO modulated neutrophil, lymphocyte, monocyte and eosinophil counts (Table 3). Although monocyte and eosinophil counts showed some variation, this could not be said to be due to the drug. The neutrophil count increased compared to that of the control at all time points except after 7 days of withdrawal but was not significantly different between the doses. The lymphocyte count decreased after 1 day of exposure and reached 0.95 and 1.90 mg/L after 21 days of exposure. The effects on both were obvious beginning on day 7 (neutrophil: F = 57.209, df = 4, *p* < 0.0001; lymphocytes: F = 52.735, df = 4, *p* < 0.0001) and abated after 7 days of withdrawal. Monocytes and basophils were not affected.

**Table 3:** White blood cell differentials of *Clarias gariepinus* (*n* = 6) exposed to propranolol

Parameter	Conc. (mg/L)	Duration (day)				
		1	7	14	21	7-w
Neutrophil (%)	Control	18.50 ± 0.50^b1^	24.00 ± 4.00^b1^	24.00 ± 4.00^b1^	21.5 ± 2.50^b1^	17.50 ± 2.50^a1^
	0.63	25.00 ± 5.00^a2^	37.00 ± 3.00^a3^	32.50 ± 2.50^a3^	17.50 ± 2.50^a3^	16.50 ± 1.50^a1^
	0.95	22.50 ± 2.50^b1^	37.50 ± 7.50^a2^	42.50 ± 2.50^a2^	32.50 ± 2.50^a2^	20.00 ± 0.00^a1^
	1.90	17.00 ± 1.00^b1^	37.50 ± 2.50^a2^	32.50 ± 2.50^a2^	39.00 ± 0.50^a2^	20.50 ± 4.50^a1^
Lymphocyte (%)	Control	79.00 ± 1.00^a1^	72.50 ± 2.50^a1^	73.50 ± 3.50^a1^	77.00 ± 3.00^a1^	79.00 ± 1.00^a1^
	0.63	71.50 ± 3.50^a2^	61.50 ± 3.50^b1^	67.00 ± 3.00^b1^	81.00 ± 1.00^a2^	82.50 ± 2.50^a2^
	0.95	75.00 ± 5.00^a2^	58.50 ± 6.50^b1^	56.00 ± 4.00^b1^	67.00 ± 3.00^b1^	77.00 ± 3.00^a2^
	1.90	80.50 ± 0.50^a2^	62.50 ± 2.50^b1^	66.50 ± 3.50^b1^	58.50 ± 0.50^b1^	78.50 ± 3.50^a2^
Monocyte (%)	Control	2.00 ± 0.00^a1^	1.50 ± 0.50^a1^	1.00 ± 0.00^a1^	1.00 ± 0.00^a1^	2.00 ± 0.00^a1^
	0.63	1.50 ± 0.50^a1^	1.50 ± 0.50^a1^	0.50 ± 0.50^a1^	1.00 ± 1.00^a1^	0.50 ± 0.50^a1^
	0.95	1.00 ± 1.00^a1^	2.50 ± 1.50^a1^	0.50 ± 0.50^a1^	0.50 ± 0.50^a1^	1.00 ± 1.00^a1^
	1.90	1.00 ± 0.00^a1^	0.00 ± 0.00^a1^	1.00 ± 1.00^a1^	1.50 ± 0.50^a1^	0.50 ± 0.50^a1^
Basophil (%)	Control	0.50 ± 0.50^a1^	1.00 ± 0.00^a1^	1.00 ± 1.00^a1^	0.00 ± 0.00^a1^	0.50 ± 0.50^a1^
	0.63	0.50 ± 0.50^a1^	0.00 ± 0.00^a1^	0.00 ± 0.00^a1^	0.00 ± 0.00^a1^	0.00 ± 0.00^a1^
	0.95	0.50 ± 0.50^a1^	1.00 ± 1.00^a1^	0.50 ± 0.50^a1^	0.00 ± 0.00^a1^	1.50 ± 1.50^a1^
	1.90	1.00 ± 1.00^a1^	0.00 ± 0.00^a1^	0.00 ± 0.00^a1^	0.00 ± 0.00^a1^	0.50 ± 0.50^a1^
Eosinophil (%)	Control	0.00 ± 0.00^a1^	1.00 ± 1.00^a1^	0.50 ± 0.50^a1^	0.50 ± 0.50^a1^	1.00 ± 1.00^a1^
	0.63	1.50 ± 0.50^a1^	0.00 ± 0.00^a1^	0.50 ± 0.50^a1^	0.50 ± 0.50^a1^	0.50 ± 0.50^a1^
	0.95	1.00 ± 1.00	0.50 ± 0.50	0.00 ± 0.00	0.00 ± 0.00	0.50 ± 0.50
	1.90	0.50 ± 0.50	0.00 ± 0.00	0.50 ± 0.50	0.50 ± 0.50	0.00 ± 0.00

Values with different small letter alphabet superscript were significantly different for drug concentrations, while values with different numeric superscript across a row were significantly different for durations (*p* < 0.05). 7-w = 7 day withdrawal

### Biochemical parameters

**Fig. 3:**
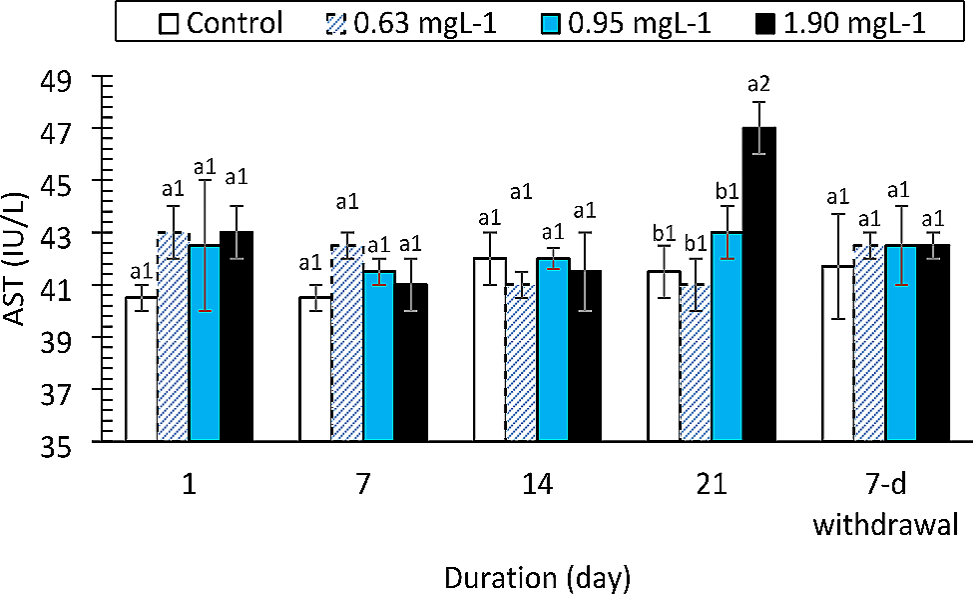
Aspartate aminotransferase activity in *Clarias gariepinus* (*n* = 6) exposed to propranolol. Bars with different alphabet label for a given day were significantly different for drug concentrations, while bars with different numeric label across were significantly different for durations (*p* < 0.05)

**Fig. 4:**
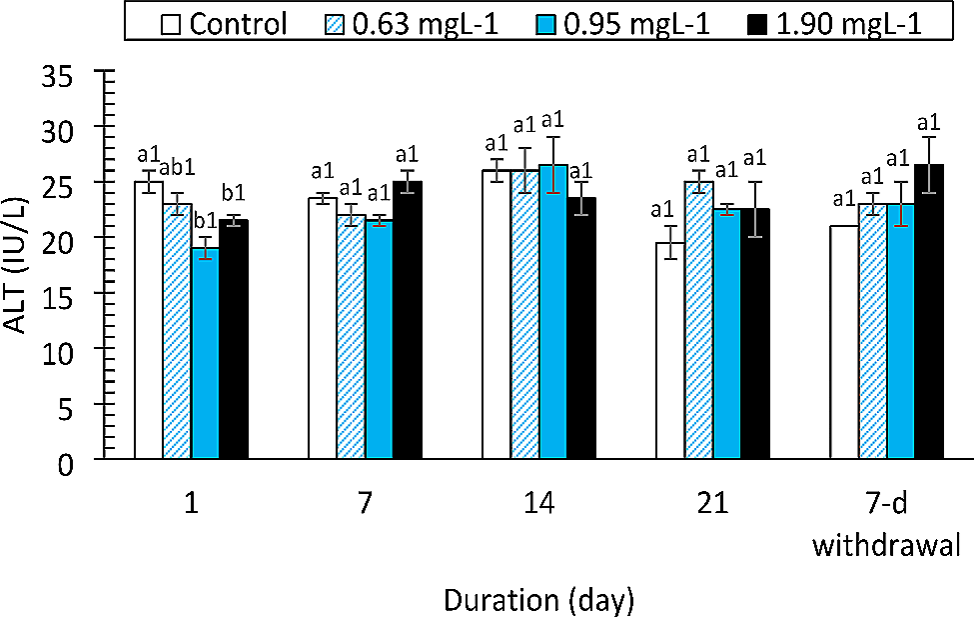
Alanine aminotransferase activity in *Clarias gariepinus* (*n* = 6) exposed to propranolol Bars with different alphabet label for a given day were significantly different for drug concentrations, while bars with different numeric label across were significantly different for durations (*p* < 0.05)

**Fig. 5:**
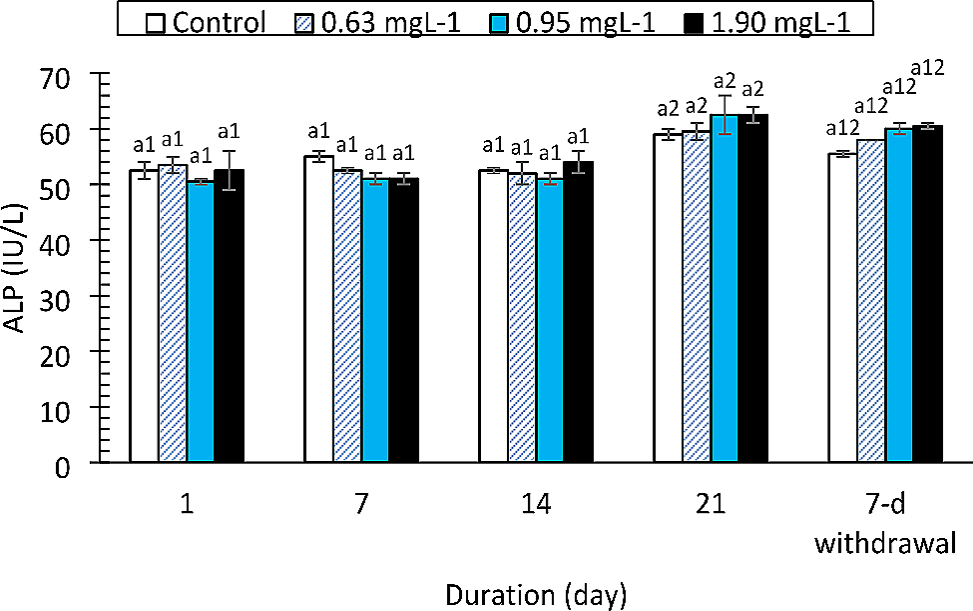
Alkaline phosphatase activity in *Clarias gariepinus* (*n* = 6) exposed to propranolol. Bars with different alphabet label for a given day were significantly different for drug concentrations, while bars with different numeric label across were significantly different for durations (*p* < 0.05)

**Fig. 6:**
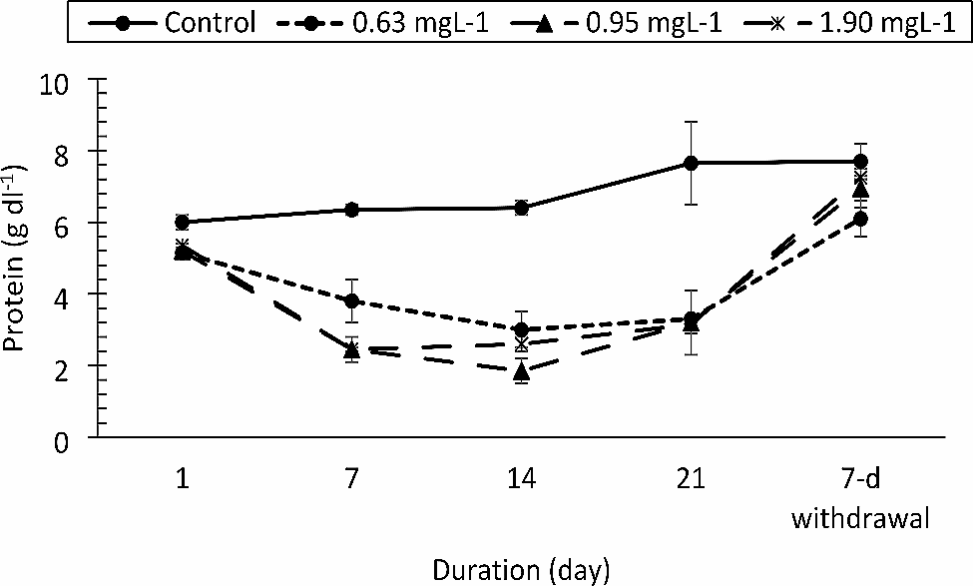
Total protein concentration of *Clarias gariepinus* (*n* = 6) exposed to propranolol

**Fig. 7:**
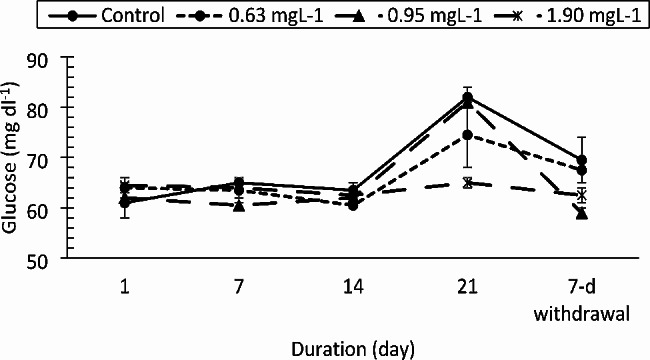
Glucose concentration of *Clarias gariepinus* (*n* = 6) exposed to propranolol

The AST, ALT and ALP enzymes responded differently to the drug. The AST values (Fig. 3) increased significantly only at the 1.90 mg/L PRO concentration compared to those of the control on day 21 (F = 11.959, df = 3, *p* < 0.0001). The change in AST was strongly affected by exposure duration (F = 25.332, df = 4, *p* < 0.0001). ALT activity (Fig. 4) was not altered by the drug at the concentrations tested (F = 3.050, df = 3, *p* = 0.039, η_p_^2^ = 0.186). However, there was a significant decrease in ALT activity compared to that in the control group on day 1 of the study (F = 83.362, df = 4, *p* < 0.0001). ALP activity was not affected by the drug (F = 1.903, df = 3, *p* = 0.145) except at 0.95 and 1.90 mg/L on day 21 of exposure and at the 7-day withdrawal of the drug (Fig. 5). Total protein levels (Fig. 6) were significantly reduced by the drug (F = 137.334, df = 3, *p* < 0.0001) in a manner dependent on concentration and exposure duration (F = 124.046, df = 4, *p* < 0.0001) but returned to normal after the 7-day recovery. The glucose concentration was lower in the fish treated with the drug than in the control fish and significantly decreased on day 21 and day 7 after withdrawal (Fig. 7).

## Discussion

Fish in their natural habitats are extremely sensitive to environmental pollutants. The HSI and CF are morphological indices of fish health [21, 37]. The body weight of the exposed fish decreased but was not significantly different from that of the control fish in the present study. The decrease in body weight observed may be attributed to stress resulting from prolonged exposure to the drug. CF and HSI were not significantly impacted by the drug, although CF seemed to gradually decline as the experiment progressed.

After exposure to pharmaceuticals, the health status of fish can be determined by changes in hematological parameters [22, 25]. Red blood cell (RBC) and hemoglobin (Hb) levels in the PRO-exposed fish were lower than those in the control group beginning on day 7 of exposure. RBC levels were significantly lower only than those in the control at 21 days postexposure, while Hb levels significantly decreased in the fish exposed to all concentrations of PRO except on day 1. Hemoglobin is carried by red blood cells to provide oxygen to all tissues and organs. Research on a variety of vertebrates, including fish, has revealed extremely distinctive erythropoietic responses to external stress [38]. Significant decreases in RBC and Hb in the PRO-exposed fish are signs that erythrocyte production is impaired. It is possible that this change in function of red blood cells caused anemia in the fish. The inhibition of hemoglobin synthesis may have been responsible for the significant decrease in hemoglobin levels in this study. This might have led to a reduction in the supply of oxygen in the fish. Pharmaceuticals have been shown to have comparable effects on fish RBC and Hb levels according to other authors [14, 39, 40]. When *Oncorhynchus mykiss* were exposed to verapamil, Velisek et al. [20] observed a significant decrease in RBC. After chloramphenicol was administered to African catfish *C. gariepinus* at a sublethal dose, Nwani et al. [14] noticed a significant change in RBC and Hb values. Additionally, Ogueji et al. [22] reported that *C. gariepinus* exposed to subchronic diazepam concentrations experienced significant decreases in red blood cell and hemoglobin counts. Our present findings are in line with those of Melefa et al. [23], who reported that clotrimazole exposure significantly reduced RBC and Hb levels in *C. gariepinus*. Like our findings, Sayed et al. [41] also reported a decrease in RBC and Hb in *C. gariepinus* exposed to dexamethasone. The packed cell volume (PCV) was lower in the groups exposed to PRO throughout the duration of the study. This decrease may be due to anemia [21]. The PCV values in the fish exposed to PRO in this study decreased compared to those in the control at 1 d, 7 d and 21 d but were not significantly different between the two doses. This finding is consistent with the findings of Nwani et al. [15] who treated *C. gariepinus* with albendazole. The PCV values of rainbow trout were significantly decreased. Additionally, according to Li et al. [39], *Oreochromis niloticus* juveniles exposed to various verapamil concentrations exhibited significantly decreased PCV values.

Fish red cell indices (MCV, MCH, and MCHC) are essential diagnostic tools for anemia [22, 42]. Different types of anemia are indicated by changes in these blood indices (macrocytic or microcytic). Although the values of these red blood cell indices varied in this study, these variations were not statistically significant. On the other hand, several authors have documented how medications affect red cell indices. According to Ogueji et al. [22], prolonged exposure of *C. gariepinus* juveniles to various diazepam concentrations resulted in significant increases in the MCV, MCH and MCHC. Additionally, *Cirrhinus mrigala* (Indian Carp) exposed to ibuprofen exhibited significant increases in MCH and MCV values [43]. When *C. gariepinus* was exposed to clotrimazole at 7.76, 3.89, or 1.94 mg/L, significantly greater levels of MCHC and MCH were observed [23]. The findings for the red cell indices in this study are consistent with those of other authors for fish exposed to different contaminants (44, 39 and 14). These authors noted that variations in red cell indices were not significantly different. According to the present study, drug-induced anemia might be normocytic in nature.

In the present study, white blood cell counts increased significantly in the fish groups exposed to PRO throughout the duration of the study. Changes in white blood cells have an impact on a fish’s ability to maintain a healthy immune system [45]. The significant increase in WBC count in the PRO-exposed group may be an immune system reaction to the toxicity induced by the drug. Other authors have noted comparable elevated WBC levels following the exposure of various fish species to drugs. When *C. gariepinus* juveniles were exposed to chloramphenicol at sublethal concentrations, a significant increase in WBC count was found. When *Oreochromis niloticus* were exposed to verapamil, a significant increase in WBC was also observed [46]. A significant increase in WBC counts was also noted in rainbow trout (*Oncorhynchus mykiss*) exposed to carbamazepine [30].

The integrity of an animal’s immune system can be determined by variation in WBC differential counts [47]. The neutrophil count increased but the lymphocyte count decreased, whereas the monocyte, basophil and eosinophil counts were not affected. Like our results, Sayed et al. [41] reported a decrease in the number of large lymphocytes in *C. gariepinus* exposed to dexamethasone, but Nwani et al. [14] reported a significant increase in the lymphocyte count in *C. gariepinus* exposed to chloramphenicol. Drug-induced lymphocytosis has also been reported in *C. gariepinus* exposed to albendazole [15] and diazepam [22]. The observed increase in neutrophils in the PRO-exposed fish in this study is consistent with the findings of Barros-Becker et al. [48] in zebra fish (*Danio rerio*) exposed to oxytetracycline. Nwani et al. [14] also noted increased neutrophil counts in chloramphenicol-exposed *C. gariepinus*. Additionally, catfish exposed to diazepam had significantly greater neutrophil counts, according to Ogueji et al. [22]. On the other hand, Nwani et al. [15] reported that the neutrophil count in catfish exposed to various albendazole concentrations significantly decreased. However, they explained that this was due to the physiological reaction of the fish to the stress imposed by the drug. Our study indicated that PRO had no effect on monocytes or basophil or eosinophil cells in juvenile *C. gariepinus*. In line with these findings, Ogueji et al. [22] reported that diazepam had no appreciable impact on monocytes or eosinophils. Boomker [49] reported the absence of basophils and eosinophils in *C. gariepinus* from South African freshwater. However, Sayed et al. [49] reported the presence of eosinophils but the absence of basophils in *C. gariepinus* exposed to dexamethasone.

Our present study indicated the presence of basophils and eosinophils in *C. gariepinus* juveniles exposed to PRO, although the values were not different from those of the control. The presence of basophils and eosinophils in *C. gariepinus* juveniles exposed to albendazole, ivermectin, clotrimazole and haloperidol have been reported by different authors [15, 23, 50, 51]. Basophils are not usually very prominent in the peripheral blood of *C. gariepinus*, and their proportions are not usually significant but can be identified through staining techniques and quantification procedures [52]. Further studies may confirm whether this difference is due to quantification procedures, the age of the fish or other unknown factors in the environment. When fish species were exposed to various concentrations of pharmaceuticals, other authors also reported no appreciable differences in these WBC differentials [53, 54]. However, Gomulka et al. [55] reported that when European white fish were given the anesthetic drug propofol, these parameters were significantly lower.

The metabolism and detoxification of drugs, chemicals, and byproducts of bacterial metabolism are carried out primarily by the liver [56]. The liver is susceptible to toxic injury from xenobiotics caused by either short- or long-term exposure because of the abundant blood supply it receives [57]. Hepatotoxicity, which results in the release or leakage of liver enzymes into the bloodstream, can be due to toxicant exposure. There were changes in the ALT, AST and ALP levels in the PRO-exposed fish, but only the AST level was significantly increased. Alanine is metabolized by the cytoplasmic enzyme alanine aminotransferase (ALT), which is found primarily in the liver [58]. The enzyme ALT leaks into the extracellular space following hepatocellular injury and enters the blood [59]. An increase in plasma ALT activity indicates damage to the cell’s cytoplasmic membrane [57]. A membrane-bound enzyme known as alkaline phosphatase (ALP) is particularly effective at facilitating the hydrolysis of a phosphate group from an organic molecule at an alkaline pH [60]. It can be used to diagnose liver or bone disease, as well as tumors in these organs, when it is present in serum at high concentrations [57]. In the present study, the PRO treatment had no effect on the levels of ALT or ALP, although ALP levels were elevated on day 21. In contrast to the results of this study, [22] found that fish exposed to ibuprofen had higher levels of these enzymes. According to [61], Nile tilapia (*Oreochromis niloticus*) exposed to various concentrations of CYP exhibited an increase in alanine transaminase (ALT) levels. The levels of ALT and ALP in fish exposed to various concentrations of diclofenac were also reported to increase [62, 63]. Additionally, fish exposed to carbendazim were reported to have higher plasma ALT and alkaline phosphatase (ALP) concentrations [21]. Melefa et al. [23] also reported a concentration- and duration-dependent significant increase in the expression of these enzymes in catfish exposed to clotrimazole.

The metabolism (transamination) of aspartate is carried out by the cytoplasmic and mitochondrial enzyme aspartate aminotransferase (AST), which is found primarily in the liver, heart, skeletal muscles, kidney, erythrocytes, and other tissues [57, 58]. Increased AST levels are a sign of liver damage [64]. In this study, the AST levels in the group exposed to 1.90 mg/L of the drug increased significantly at 21 days postexposure, but not significantly different from those in any other group. This finding supported the findings of a study in which fish exposed to carbendazim had elevated AST levels [21]. Odo et al. [65] also reported that AST levels significantly increase in response to ivermectin exposure at concentrations of 0.007, 0.014, and 0.033 mg/L. This study supported the findings of Akinrotimi et al. [16]. When *C. gariepinus* was exposed to 50, 100, 150, or 200 mg/L anesthetic, a discernible increase in AST was observed. On the other hand, fish exposed to 0.1 LC_50_ of diclofenac had significantly lower AST activity [62]. In the present study, a significant decrease in protein levels was observed. This might be because the toxic effects of drugs on fish liver after exposure inhibit the synthesis of proteins [21, 66]. This decrease might be due to the metabolic use of ketoacids in the production of glucose to compensate for the high energy demands of the drug. Lower levels of total plasma protein in fish exposed to carbendazim concentrations of 0.22 and 0.43 mg/L have been reported, and these findings are consistent with the findings of this study [21]. Fish exposed to diclofenac also had lower protein levels [63]. When *Rhamdia quelen* was exposed to pharmaceuticals, a significant decrease in the total protein concentration was reported [67]. In addition, catfish exposed to clotrimazole had lower protein levels [23]. A steady decrease in protein levels in an organism exposed to xenobiotics is a sign of liver toxicity [68].

The fish in the PRO group exhibited a significant decrease in tissue glucose levels, but the values increased at 21 days postexposure in all groups, including the control group. The reduction in glucose levels observed in the PRO-exposed fish in this study indicated a disruption in the carbohydrate metabolism pathway, which may have been influenced by the decreased activity of glucose-6-phosphate in the liver or by the decreased rate of glycogen breakdown [63, 69]. In line with the results of this study, *Cirrhinus mrigala* individuals exposed to clofibric acid and diclofenac both acutely and chronically had significantly lower plasma glucose levels [70]. The increase in glucose levels after 21 days postexposure may be an indication of hyperglycemia resulting from gradual recovery of the cells from the stress induced by the drug. However, juvenile *C. gariepinus* exposed to clotrimazole had significantly greater glucose levels [23]. Additionally, juvenile *C. gariepinus* treated with carbendazim exhibited hyperglycemia [21]. Other authors have noted variations in glucose levels as signs of stress caused by toxicants in fish [14, 71].

## Conclusion

Sublethal exposure of *C. gariepinus* juveniles to PRO led to significant alterations in the biochemical and hematological parameters assessed in this study. The values of PRO detected in the various water bodies in Nigeria may be low but in view of the repeated use, and the uncontrolled disposal of hospital wastes and the expired drug, it may bioaccumulate hence the environmental relevance of our studied sublethal concentrations. This finding suggested that the drug might be toxic to fish and other aquatic organisms. Additionally, the information from this study may be useful for further environmental assessment of pharmaceutical drugs.

### Electronic supplementary material

Below is the link to the electronic supplementary material.

Supplementary Material 1

## Data Availability

The datasets are available upon request from the corresponding author.
