# Supplementary material for: Biomorphometric and hematobiochemical alterations in the juvenile african catfish Clarias gariepinus exposed to propranolol
Source: BMC Zool. 2024 Jun 20;9:11. doi: 10.1186/s40850-024-00196-x (PMC11188512; doi:10.1186/s40850-024-00196-x)
Supplement: Supplementary file 1 — Supplementary Material 1 [file 40850_2024_196_MOESM1_ESM.docx]

**Figure S1:** Mean corpuscular volume (MCV) of *Clarias gariepinus* (n = 6) exposed to propranolol.
